# Supplementary material for: Mechanisms of HIV-1 integrase resistance to dolutegravir and potent inhibition of drug-resistant variants
Source: Sci Adv. 2023 Jul 21;9(29):eadg5953. doi: 10.1126/sciadv.adg5953 (PMC11803526; doi:10.1126/sciadv.adg5953)
Supplement: Supplementary file 1 — Supplementary Note S1 to S3 Figs. S1 to S12 Tables S2 and S3 Legend for table S1 References [file sciadv.adg5953_sm.pdf]

Science Advances
AAAS

Supplementary Materials for

Mechanisms of HIV-1 integrase resistance to dolutegravir and potent inhibition of drug-resistant variants

Min Li *et al.*

Corresponding author: Dmitry Lyumkis, dlyumkis@salk.edu; Robert Craigie, robertc@niddk.nih.gov

Sci. Adv. **9**, eadg5953 (2023)
DOI: 10.1126/sciadv.adg5953

The PDF file includes:

Supplementary Note S1 to S3
Figs. S1 to S12
Tables S2 and S3
Legend for table S1
References

Other Supplementary Material for this manuscript includes the following:

Table S1

Supplementary Text

Supplementary Note 1: Modeling the fitness landscape using Potts statistical energy models built on the Stanford drug resistance DB:

A hallmark of drug resistance is the pairing of one or more primary mutations that interfere with drug binding and for which there is often a fitness cost, with one or more compensatory mutations that improves the fitness in the presence of the primary mutation(s). The association of primary with compensatory mutations is a feature of epistasis, *i.e.* the coupling of fitness effects that are not independent (*44*). The Potts statistical energy models probe the underlying relationships between protein structure, function, and fitness and, here, they provide insight into coupled primary and compensatory residue pairs that help to explain the diverse data at hand.

The population-based Potts model of the fitness landscape is defined though an expression which defines the likelihood of a sequence in a given environment, and it changes as the environment changes. It has the following functional form: $E(S) = \sum_{i<j} J^{ij}_{si\,sj} + \sum_i h^i_{si}$ where the residue-residue couplings $J^{ij}_{si\,sj}$ correspond to epistatic interactions involving compensatory constraints between residues within the protein, and the field terms h^i_{si} represent single-site biases. A parsimonious interpretation of these two terms is that the compensatory constraints encoded in the couplings, like those that enforce correct folding, are "intrinsic" and operate in all the environments, while the field terms correspond to "external" selection pressures, like the immune response and drug selection pressure. Fitness is always defined relative to an environment, and in this decomposition of the fitness, different environments (for instance different drug regimens) will correspond to different parameterizations of only the field terms.

Our Potts model is fit to drug-exposed sequences (mainly exposed to **RAL**, with a minority exposed to **EVG**, **CAB**, **DTG**, and **BIC**), and therefore models a global drug-experienced fitness environment for the virus, reflecting its survival as it evolves across hosts given combinations of these drugs. As anticipated by our interpretation of the physical meaning of the fields and couplings presented above, we have confirmed that starting with the drug experienced model, we can transform it to model the drug-naïve fitness landscape which accurately predicts mutant frequencies of a drug-naïve sequence dataset by modifying only the field terms of the drug experienced model. Both this drug-experienced model and the drug-naive model predict the respective pairwise mutant frequencies in each dataset with a maximum of 1% error, and on average only 0.07% error, even though the pairwise mutant frequencies of the drug-naïve dataset are up to 53% different from the drug-experienced mutant frequencies. These two models share the same (intrinsic) coupling terms, but have different (external) field terms.

Double-mutant-cycles, defined as $\Delta\Delta E$ in Methods, equal the fitness effect of the double mutant minus the sum of the two single mutant fitness effects; the external field terms of the Potts model cancel out of this computation. Therefore, the double-mutant-cycle values have the special property that they reflect epistatic constraints within the enzyme present in all environments, and their values are the same for both the drug-experienced and derived drug-naïve model described above. This provides the rationale for our using the predictions of double-mutant-cycles to estimate intrinsic epistatic effects that are independent of drug exposure, even though our Potts model is fit to data exposed mainly to **RAL**. We perform our inference and computations using the drug-experienced dataset, because it has more extensive sequence variation than the drug-naïve data, giving a more accurate model parameterization of the intrinsic epistatic interactions.

The results of the double mutant cycle analysis (**Fig. 2D**) show the non-additive effects (cooperativity) between compensatory pairs of mutations in the protein sequence. The double

mutant cycle values DDE depend only on the Potts model coupling terms $I(i_a,j_b)$, not the field terms. The coupled mutation pairs involving residues at positions 138, 140, and 148, which are the central focus of the current work, are among those with the strongest epistatic couplings. While the majority of drug experienced data present in the Stanford DB are derived from patients who are treated with 1st generation INSTIs, the couplings derived from this dataset reflect epistatic effects due to intrinsic constraints on fitness induced by the structure and function of the enzyme, independent of the application of a specific drug. This reinforces the view that the epistatic interactions among mutations at positions (138,140,148) that are captured by the Potts model reflect fitness constraints imposed by the structure and function of the protein, independent of a specific drug.

Supplementary Note 2. Correlation between FEP-based binding affinities and experimental measurements of EC_{50} in cells

We show that experimental EC_{50} values correspond closely to the calculated dissociation constant K_D. As seen in **table S2**, based on the FEP calculations, we estimate that with the introduction of the third mutation, G140A, the binding affinities of **DTG** and **4d** are reduced by 1.0 and 0.3 kcal/mol (for dominant conformers), respectively, which correlates well with the experimentally determined EC_{50} values from virology assays. This result suggests that the atomistic free energy model captures the trend of the drug resistance mutation of the IN to the INSTIs, and such techniques may further improve our understanding of the drug resistance mutations to the INSTIs.

Supplementary Note 3. 4d binds more strongly to the E138K/G140A/Q148K triple mutant when the adenine base forms a stacking interaction with the ligand

A comparison of the Apo structure (PDB: 6PUT) (*15*) to the INSTI-bound structures of the Intasome shows that the difference is largely confined to the conformation of the terminal adenosine (dA21) of the vDNA, which can adopt two distinct conformations in the active site of INSTI-bound structures: (i) one in which the dA21 base forms a stacking interaction with the bound INSTI (stacked) and (ii) one in which the base extends into the inter-subunit space (non-stacked). Both structures are distinct from the conformation of vDNA in the apo cryo-EM structure. Using the thermodynamic cycle shown in **Fig. 5G**, we can gain insight into the relative binding affinities for the ligands **DTG** and **4d** to the stacked and non-stacked conformations using as constraints the experimental EC_{50} values (estimate of the dissociation constant K_D) and stacking vs. non-stacking ratios estimated from the cryo-EM densities. There are two constraint equations, which can be used to solve for the binding strength of **DTG** or **4d** to the stacked form of the complex ΔG^S and to the non-stacked form of the complex ΔG^{NS}. According to the thermodynamic cycle shown in **Fig. 5G**, $\Delta\Delta G^{S\rightarrow NS} \equiv \Delta G^{NS} - \Delta G^S = \Delta G^{S\rightarrow NS}_{complex}$, which means that we can estimate the difference in the ligand binding free energy towards the stacked vs. non-stacked conformations $\Delta\Delta G^{S\rightarrow NS} = \Delta G^{NS} - \Delta G^S$ from the knowledge of $\Delta G^{S\rightarrow NS}_{complex}$. Because $\Delta G^{S\rightarrow NS}_{complex} = \frac{1}{\beta} ln \frac{P^S_{com}}{P^{NS}_{com}}$, the ratio of the occupancies of the stacked vs. non-stacked complex states $\frac{P^S_{com}}{P^{NS}_{com}}$ can be obtained from experiments. **Table S3** summarizes the experimental constraints and shows that **4d** binds to KAK in both non-stacked and stacked conformers more strongly than **DTG** from the estimated binding free energies. Furthermore, the binding free energy difference between **4d** and **DTG** to E138K/G140A/Q148K is more negative (-2.1 kcal/mol) in the stacked conformer than in the non-stacked conformer (-0.9 kcal/mol), indicating that the stacked terminal adenine in

E138K/G140A/Q148K helps maintain bound **4d** in its preferred position, and contributes more to the greater potency of **4d** than does the non-stacked conformation.

Supplementary Figures

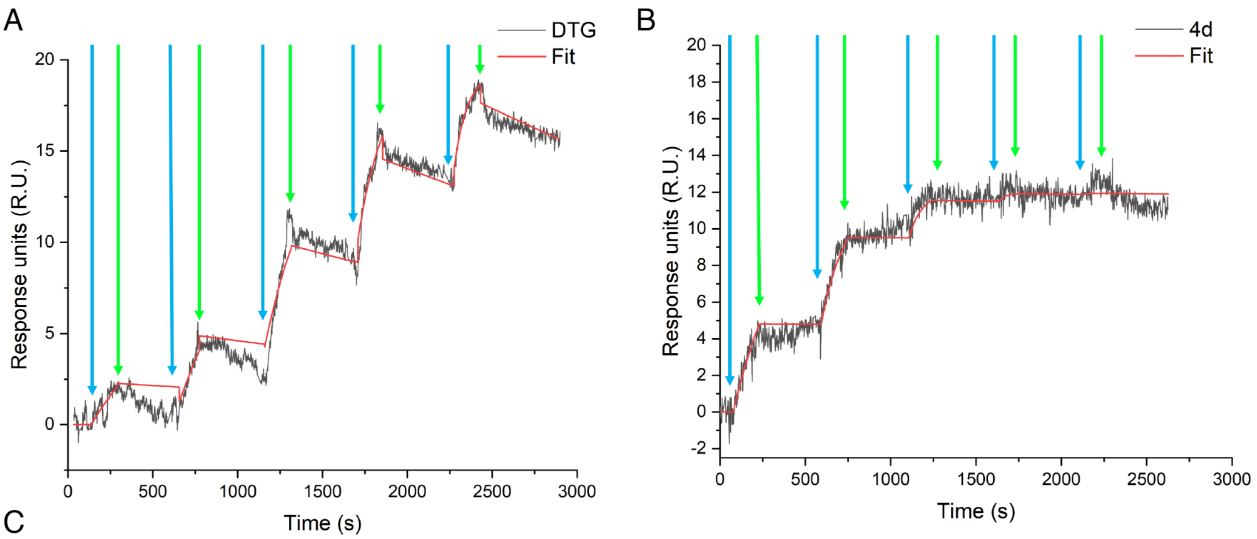

C

Drug	K_{on}	K_{off}	K_D (K_{off}/K_{on})
DTG	2.22 x 10^4 $M^{-1}s^{-1}$	6.67 x 10^{-5} s^{-1}	3.0 nM
4d	1.088 x 10^5 $M^{-1}s^{-1}$	1.637 x 10^{-6} s^{-1}	0.015 nM
	~ 5 x higher	~ 40 x lower	~ 200 x lower

Figure S1: **Surface plasmon resonance (SPR) analyses of interactions of DTG and 4d with WT PFV intasomes.** (**A**) sensorgram for **DTG**. (**B**) sensorgram for **4d**. In both panels A-B, blue arrows indicate injection time points of INSTIs into PFV intasomes followed by association steps (ascending curves). Green arrows indicate the switch to dissociation steps (descending curves) as the complexes are washed with running buffer in the absence of the inhibitor. Note that steeper descending curves observed in (**A**) vs (**B**) indicate faster dissociation of **DTG** vs **4d** from PFV intasomes. Furthermore, while identical concentrations of **DTG** and **4d** were tested, the saturation of the inhibitor-intasome complexes was reached in (**B**) but not (**A**). For each drug, the K_{on} and K_{off} rates are obtained from multiple titration points. Taken together, both kinetic and thermodynamic equilibrium analyses indicate that **4d** binds to PFV intasomes with a higher affinity than **DTG** due primarily to the slower dissociation rate of **4d** vs **DTG**. (**C**) Kinetic parameters obtained from SPR assays.

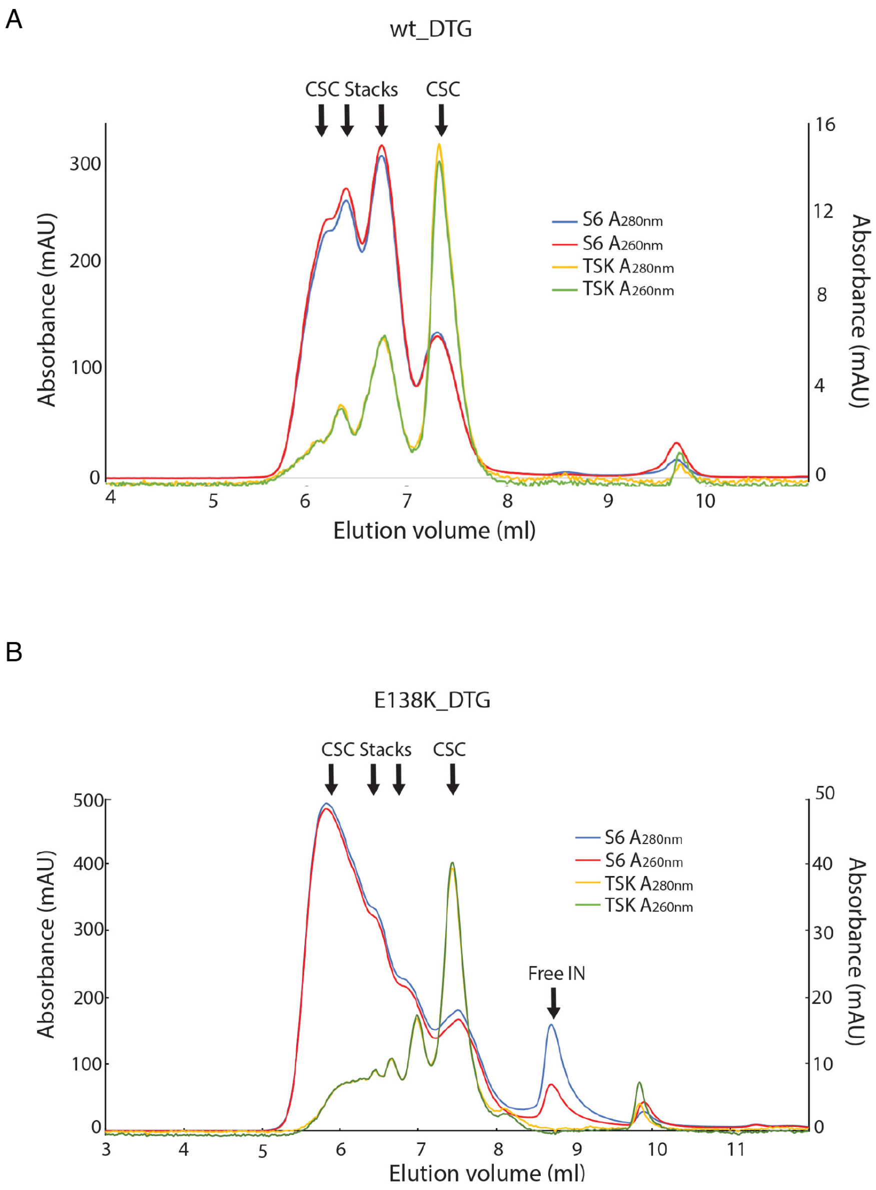

Figure S2: Purification of mutant intasomes. WT (**A**) or mutant (**B**) CSC intasomes assembled with bound DTG were first separated on a Superose 6 column. After the initial separation, partially purified mutant intasomes were subjected to a size exclusion purification step using a TSKgel Ultra HPLC column (red and blue). The profiles show a substantial amount of residual size heterogeneity, which is dominated by larger species that appeared to be proto-intasome oligomeric stacks, which contain multiple copies of vDNA and have been reported previously (*15*, *28*). Discrete intasome species eluted between 7.4 and 8.2 mL and were collected for another round of size exclusion chromatography. Reinjection of the eluted complexes onto the same TSKgel Ultra HPLC column (yellow/green curve) showed that most of the larger proto-intasome stacks were removed, yielding dodecameric intasome species for structural studies. The profiles show here are representative of the WT (**A**) or the E138K (**B**) intasome with DTG bound. The other mutant intasome assemblies exhibited similar profiles.

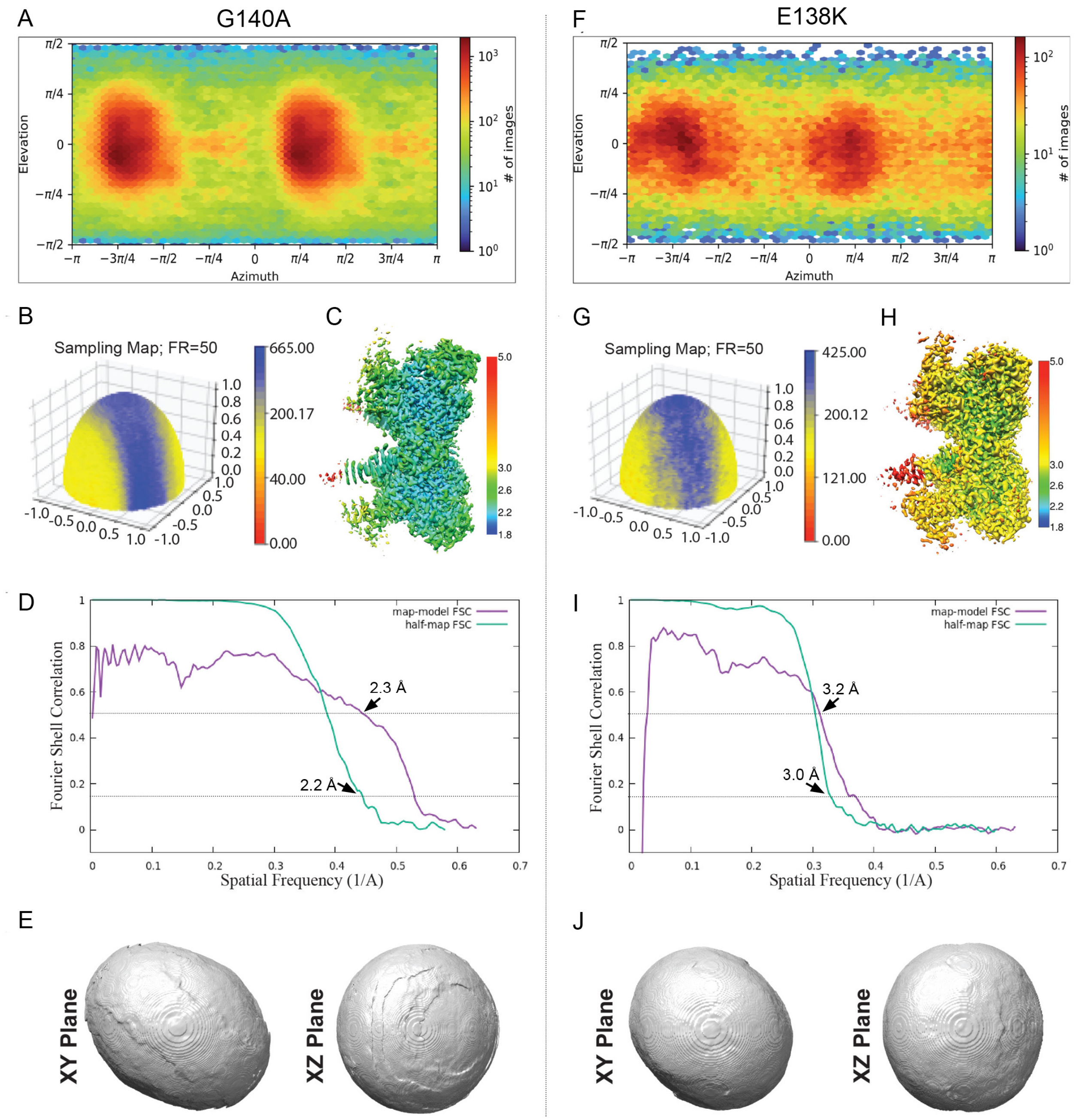

Figure S3: Cryo-EM validation of the highest resolution G140-DTG and the lowest resolution E138K-DTG intasome reconstructions. Validations metrics are displayed for (**A-E**) the G140A intasome with DTG bound or (**F-J**) the E138K intasome with DTG bound. (**A,F**) Euler angle plot showing the distribution of projection orientations used in the cryo-EM reconstruction. (**B,G**) Surface sampling plot of the Fourier voxel sampling derived from the Euler angle distribution plot, calculated with a Fourier radius set to 50 voxels and imposing C2 symmetry. The sampling compensation factor (SCF) is indicated (*73*, *74*). (**C,H**) Cryo-EM reconstruction colored by local resolution. (**D,I**) Fourier shell correlation (FSC) curves derived from half-map and map-to-model reconstructions, with FSC cutoffs 0.143 and 0.5 indicated, respectively, as well as nominal resolution values. (**E**,**J**) 3DFSC (*72*, *88*) shown as an isosurface and thresholded using a value of 0.5 with two perpendicular planar views.

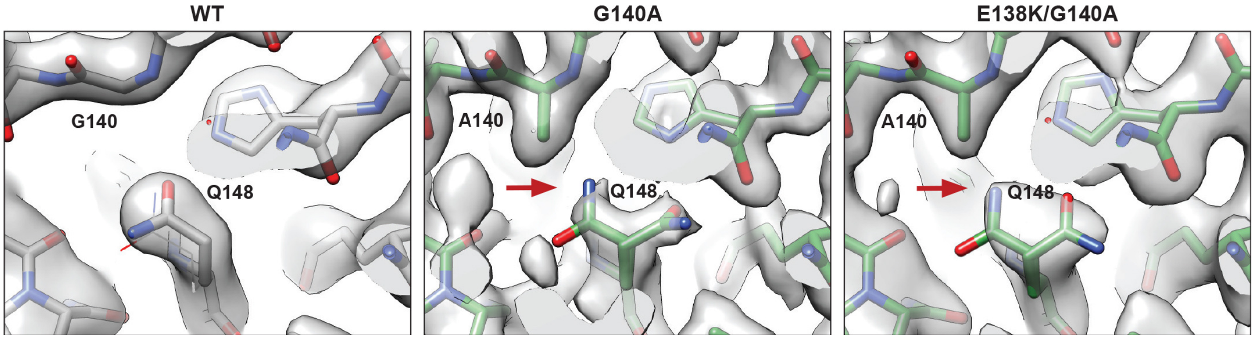

Figure S4: Rotameric configurations of Q148 in the presence of the G140A mutation and the E138K/G140 Double mutation. Cryo-EM density and the atomic models are shown for structures of WT, G140A, and E138K/G140A intasomes bound to **DTG**. Only one rotamer of Q148 is evident for the WT intasome, whereas two rotameric conformations can be modeled for G140A and E138K/G140A mutant intasomes. There is a preference for the alternative conformation of Q148 due to weak steric clashes (indicated by red arrows) if A140 is present. Residues at positions 140 and 148 are labeled.

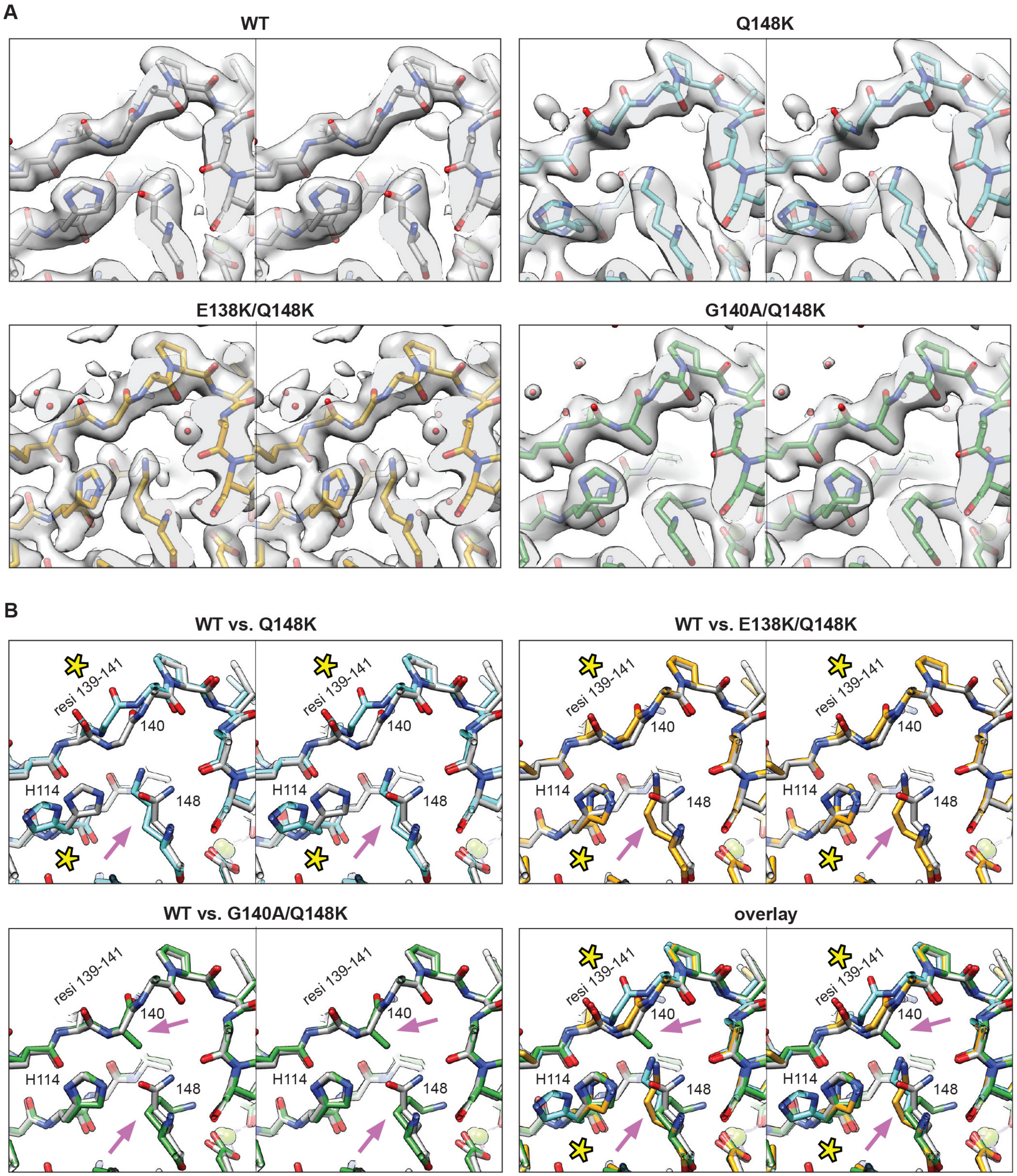

Figure S5: Changes to backbone residues 139-141 and the sidechain of H114 if the Q148K mutation is present. (**A**) Experimental cryo-EM density and atomic models of the region surrounding residue 148 are shown in stereo for structures of WT, Q148K, E138K/Q148K, and G140A/Q148K intasomes bound to **DTG**. (**B**). Overlays of atomic models showing the same region displayed in panel A. In comparison to WT, the backbone shift is readily apparent in the Q148K structure, and there is a small shift in the E138K/Q148K structure. The reconfiguration of the rotameric position of H114 is apparent in Q148K structure, whereas only a minor rotation is apparent in E138K/Q148K structure. Residues at positions 114 and 148 are labeled.

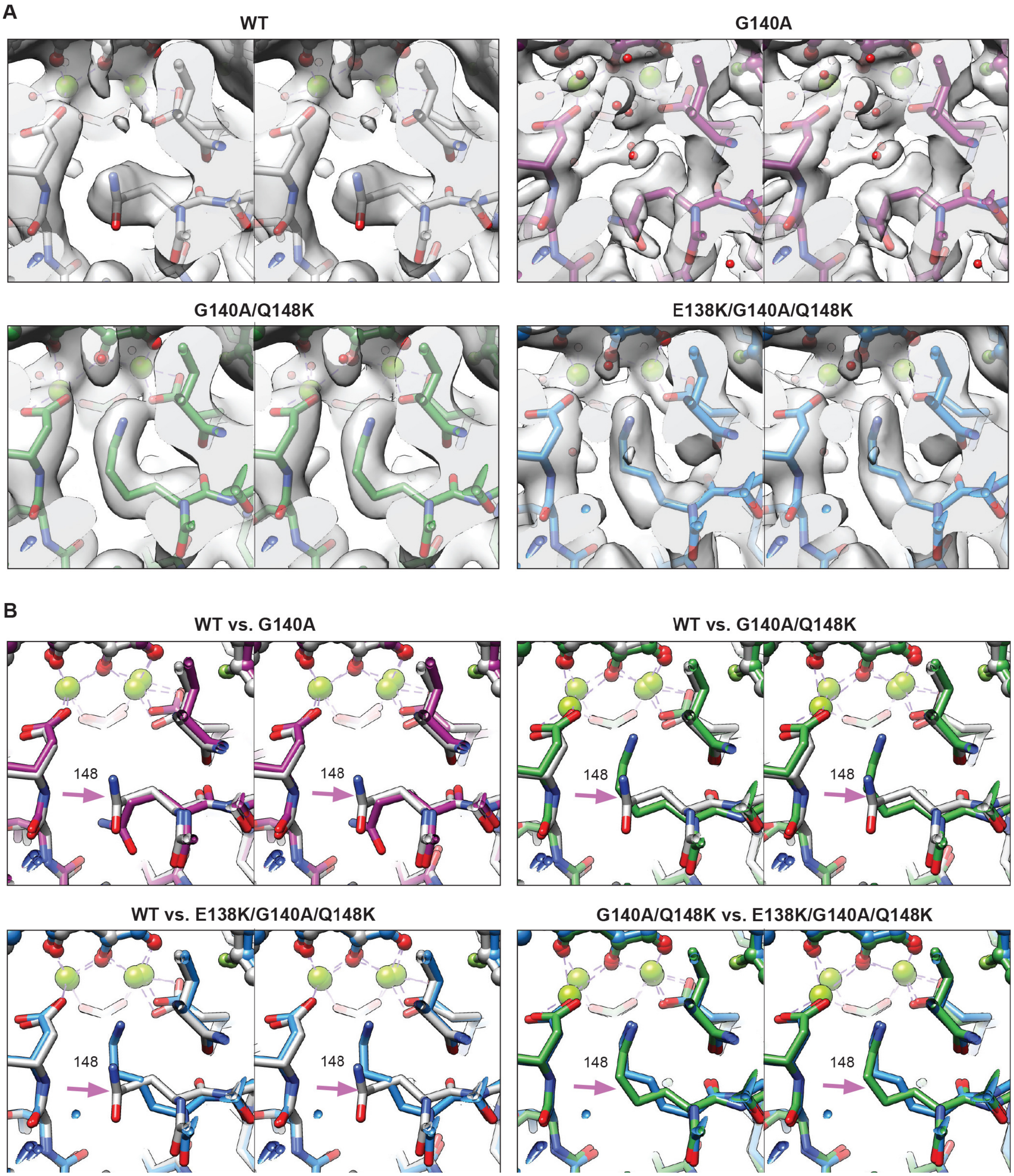

Figure S6: Alterations in the conformation of the residue at position 148 in response to the G140A mutation. (**A**) Experimental cryo-EM density and the atomic models centered around residue 148 are shown in stereo for structures of WT, G140A, E138K/Q148K, and G140A/Q148K intasomes bound to **DTG**. (**B**). Overlays of atomic models, showing the same region displayed as in panel A. In comparison to WT, the conformational change of Q148 is apparent if G140A is present. Furthermore, if the residue at position 148 is a Lys, the presence of the G140A mutation leads to a reconfiguration of the Lys sidechain to position that is closer to the Mg^{2+} ions, as shown in the structures of G140A/Q148K and E138K/G140A/Q148K. Position 148 is labeled. Red arrows indicate weak steric clashes.

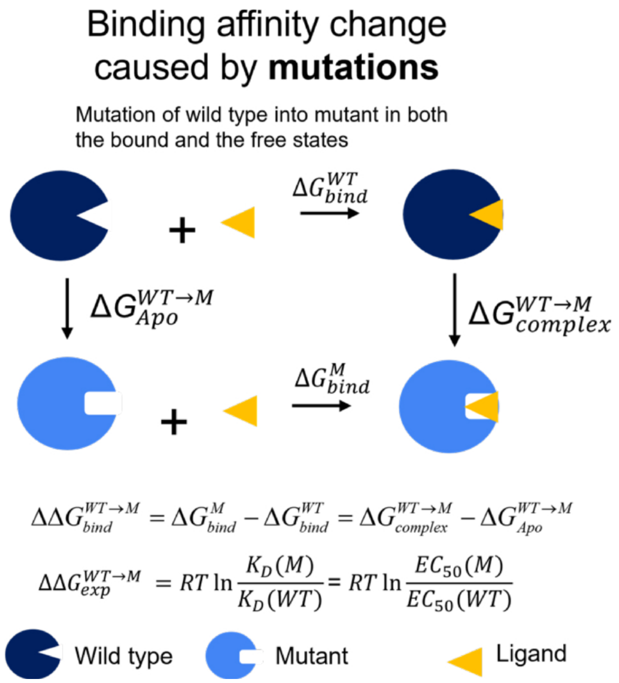

Figure S7: **Thermodynamic cycles used for MD-based FEP simulations**. The thermodynamic cycle used for computing the changes in the binding free energy caused by the residue mutation in the binding of the ligand to the intasome. We examined the changes in the binding free energy caused by the G140A mutation in the binding of either **DTG** or **4d** to either the E138K/Q148K (KK) or E138K/G140A/Q148K (KAK) DRM intasomes by using FEP (free energy perturbation) to calculate the relative binding free energy $\Delta\Delta G_{bind}^{KK\rightarrow KAK}$ for each of the ligands. In the thermodynamic cycle, $\Delta\Delta G_{bind}^{KK\rightarrow KAK} \equiv \Delta G_{bind}^{KAK} - \Delta G_{bind}^{KK} = \Delta G_{complex}^{KK\rightarrow KAK} - \Delta G_{Apo}^{KK\rightarrow KAK}$, i.e. the relative binding free energy $\Delta\Delta G_{bind}^{KK\rightarrow KAK}$ can be obtained by the two vertical legs, instead of simulating the physical binding process.

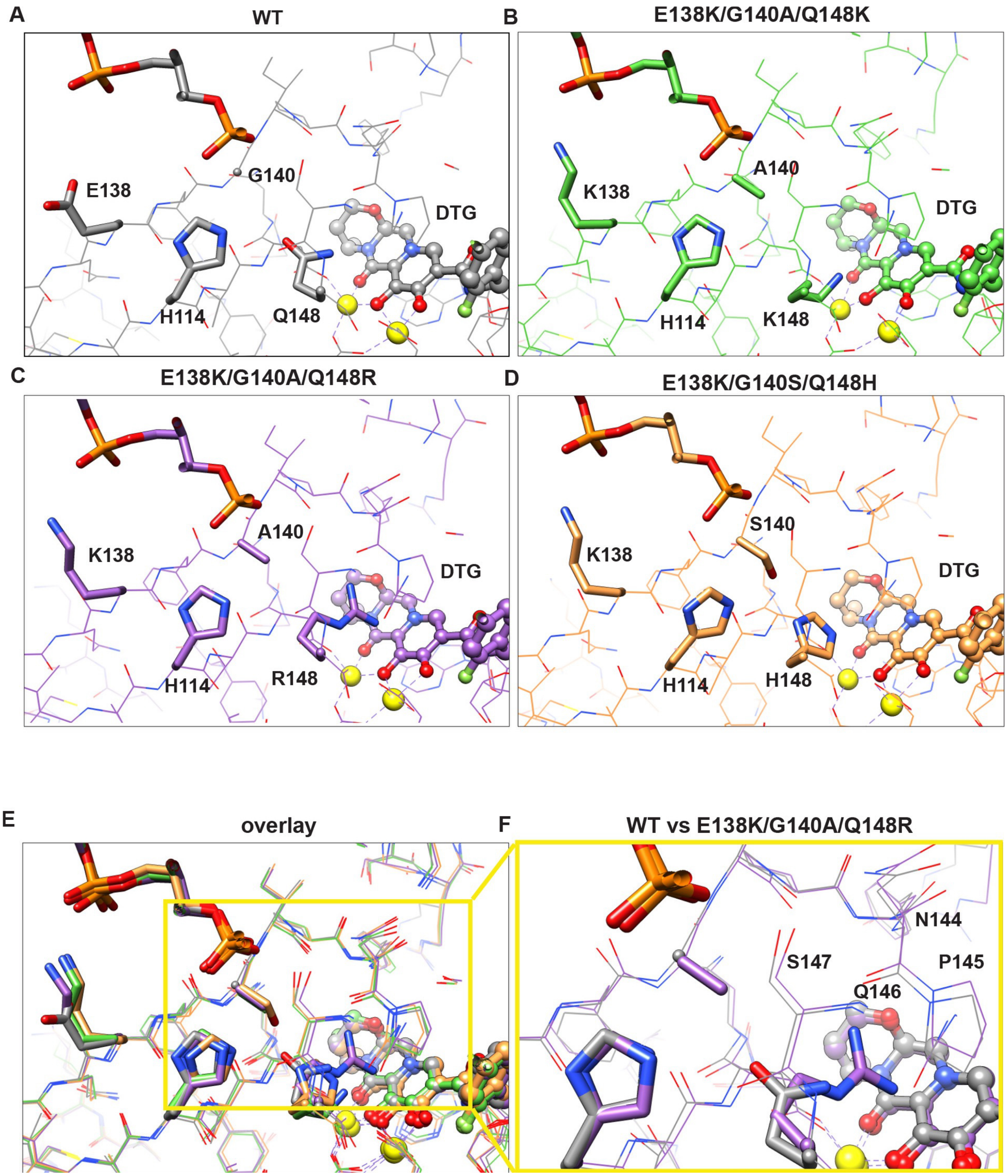

Figure S8: Comparison of the structural changes in response to Lys, Arg, and His mutations at position 148. Atomic models of the active site from high-resolution intasome structures with **DTG** bound are shown for (**A**) the WT intasome, or (**B-D**) the triple mutant variants (**B**) E138K/G140A/Q148K (**C**) E138K/G140A/Q148R, (**D**) E138K/G140S/Q148H. (**E**) An overlay of all structures in A-D. (**F**) Close-up of the region spanning N144-S147, which differs in the structure of E138K/G140A/Q148R intasome with **DTG** bound, compared with the WT intasome with **DTG** bound. The view of the active site is the same as in Figure 3.

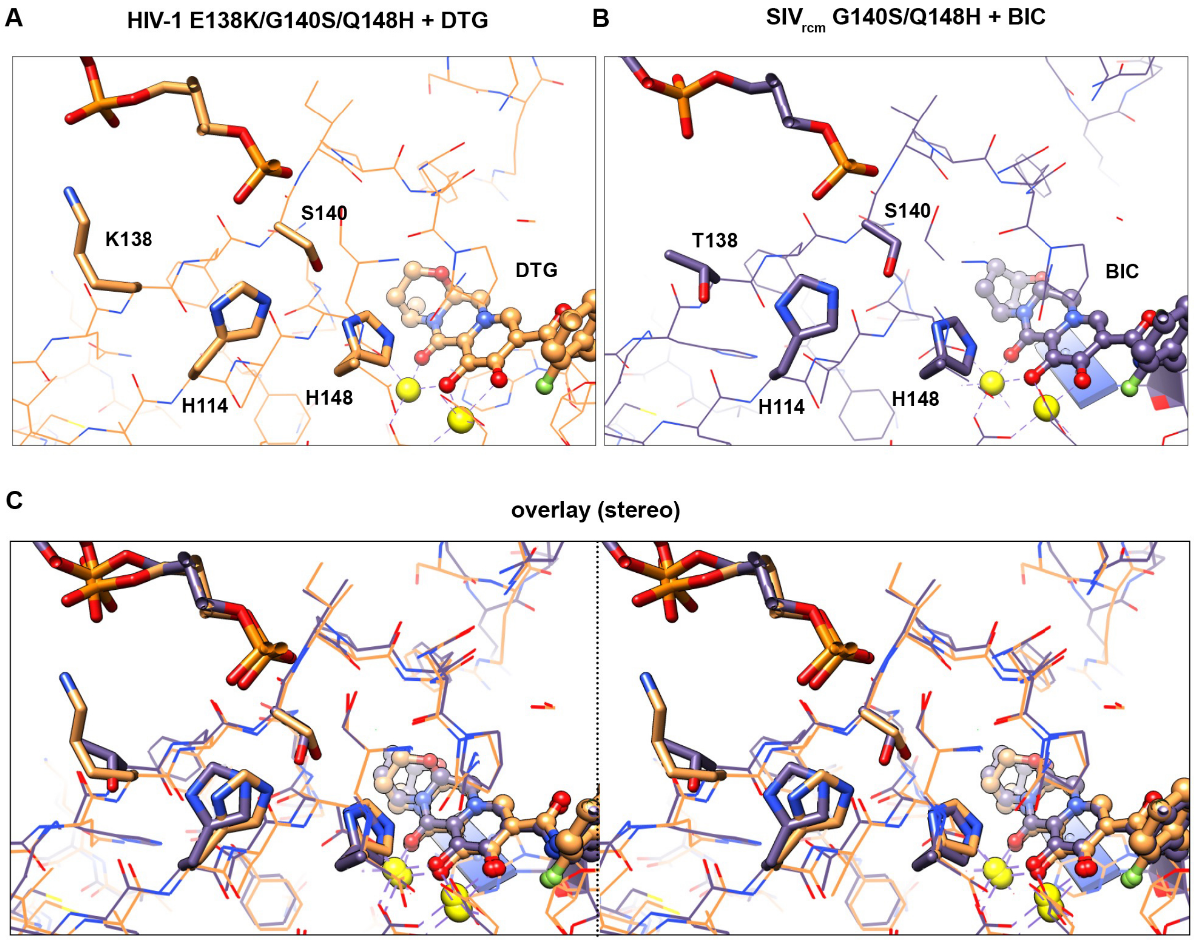

D

HIV-1	SIV_{rcm}
L63	M63
L74	I74
S119	D119
E138	T138
I141	V141
I151	V151
K156	H156
S230	G230
P233	Q233

Figure S9: Comparison between the structures of HIV-1 and SIV_{rcm} intasomes containing the G140S/Q148H mutations. Atomic models of the active site from high-resolution intasome structures are displayed for (**A**) the HIV-1 E138K/G140S/Q148H intasome with **DTG** bound, and (**B**) the SIV_{rcm} G140S/Q148H intasome with BIC bound. (**C**) An overlay of the two structures in A-B, displayed in wall-eye stereo. (**D**) A table of the distinct residues within 10 Å of the bound drugs which differ between HIV-1 and SIV_{rcm} IN. In **panels A-C**, the view of the active site is the same as in **Figure 3**.

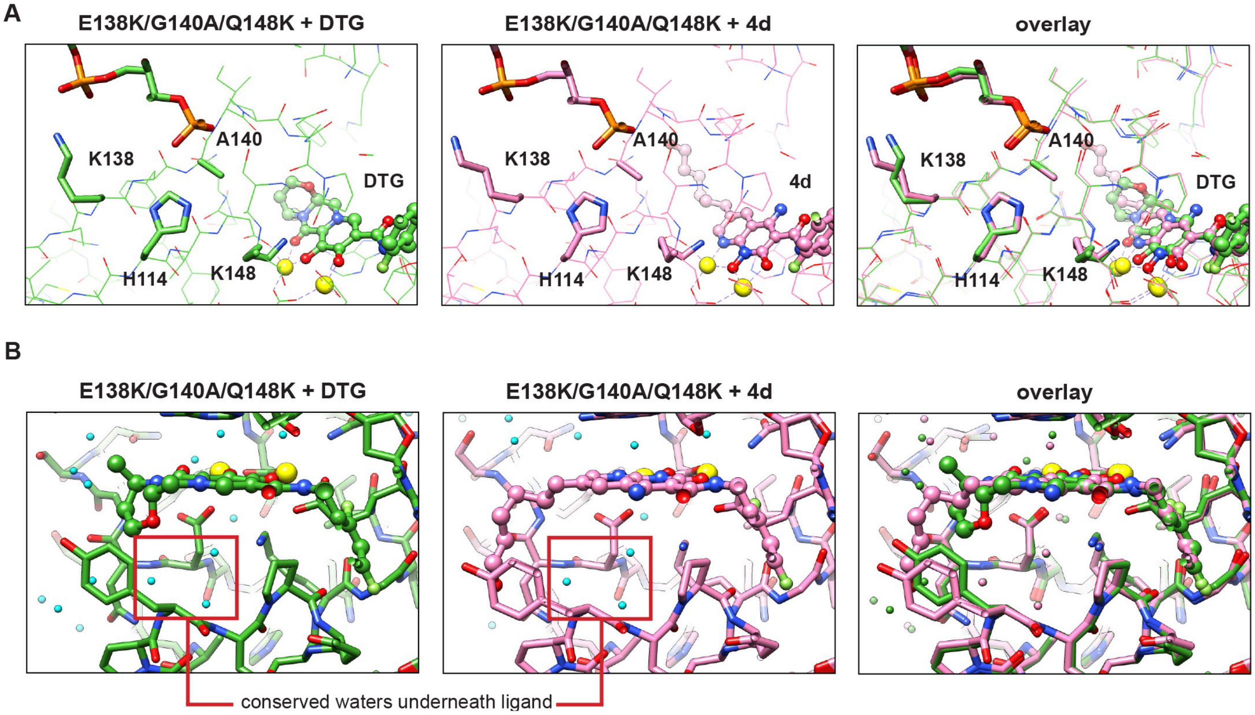

Figure S10: Comparison of the structural changes in the intasome when 4d or DTG are bound. (**A**) Atomic models of the active site from high-resolution HIV-1 intasome structures of the E138K/G140A/Q148K variant bound to either **DTG** or **4d**, with an overlay of the two at right. Only subtle changes are observed for the residues involved in the resistance mechanism. The view is identical to that in Figure 3. (**B**) Atomic models of the region surrounding the bound ligand, showing bound water molecules for each structure (cyan). An overlay of the two is at right, and the waters are colored according to its respective structure. The waters outlined in red, which reside "underneath" the ligand, are entirely conserved for the two mutants. Minor variations in the hydration shell is observed otherwise, which may arise from biological differences as well as differences in resolution.

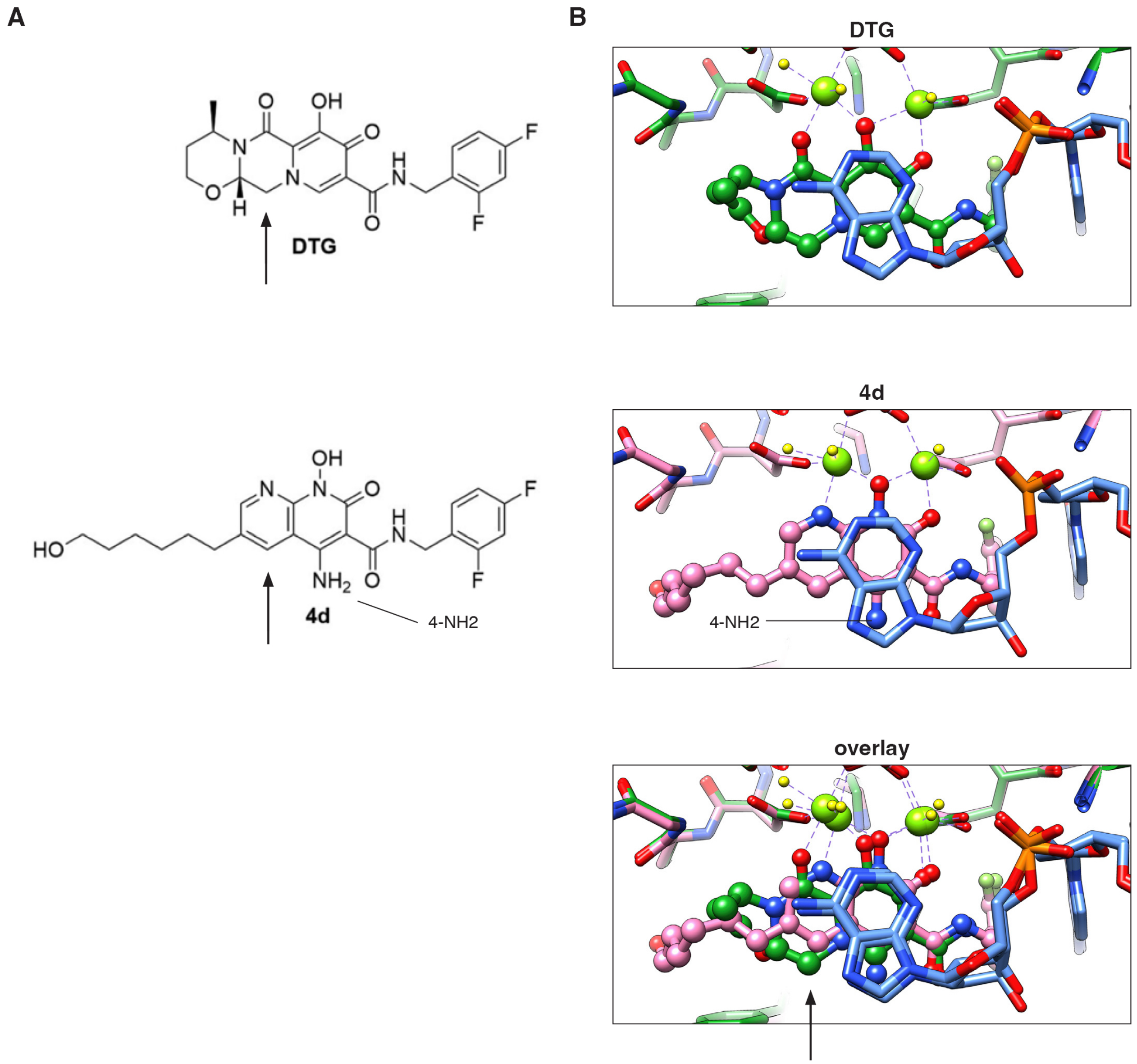

Figure S11: Comparison of the binding modes of DTG and 4d in the active site of the HIV intasome. (**A**) Chemical structures of **DTG** and **4d**. The bold arrows point to the second ring, which is not aromatic in **DTG**, but is aromatic in **4d**. The presence of two aromatic rings in **4d** may contribute to stabilizing the position of the terminal adenine. (**B**) A top-down view of the π-π stacking between the terminal adenine and the INSTI core of either **DTG** or **4d** is shown, with an overlay of the two structures at right. In the **4d** panel (middle), the 4-amino group on the compound is located underneath the five-membered ring of the adenine, which may contribute to a cation-π interaction that is specific to **4d**, but not **DTG**. In the overlay panel, the bold arrow points to the apparent shift in the position of the INSTI core, which is due to one of the electronegative atoms chelating the Mg^{2+} ion being an -OH group in **DTG** but an embedded nitrogen within the core of **4d**. This shift leads to a slight repositioning of the first ring, which can affect the strength of the π-π stacking interaction.

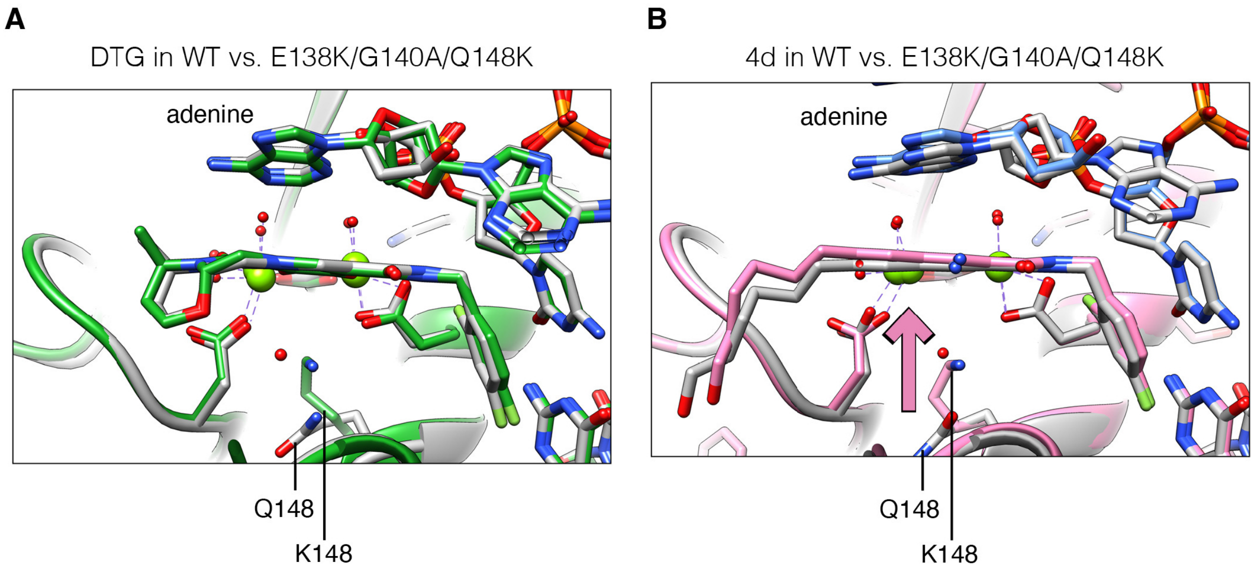

Figure S12: The ligand 4d, but not DTG, is pushed toward the terminal adenine base in the E138K/G140A/Q148K intasome. (**A**) Overlay of the atomic models of WT (gray) and E138K/G140A/Q148K (green) intasome with **DTG** bound. The ligand remains in place in the context of the triple DRM. (**B**) Overlay of the atomic models of WT (gray) and E138K/G140A/Q148K (pink) intasome with **4d** bound. The ligand is pushed “upward” in the context of the triple DRM (pink arrow).

Table S1. Cryo-EM data collection and image processing parameters.
The reader is referred to the excel sheet that is presented with the paper.

Table S2. Changes in the ligand binding free energies caused by the G140A mutation (E138K/Q148K → E138K/G140A/Q148K) for the INSTIS **DTG** and **4d** bound to IN. The first column shows the binding free energy differences from EC_{50} values ($\Delta\Delta G_{EC50}^{WT\to M} = RT \ln \frac{EC_{50}(KAK)}{EC_{50}(KK)}$) of **DTG** and **4d** against the mutant IN E138K/Q148K and E138K/G140A/Q148K estimated using FEP simulations, whereas the last column shows the experimental binding free energy differences obtained using virology assays (**Fig. 1**).

ligand	FEP Calculated ΔΔG(kcal/mol) $= \Delta G_{bind}^{KAK} - \Delta G_{bind}^{KK}$	Experimental ΔΔG(kcal/mol) $= RT \ln \frac{EC_{50}(KAK)}{EC_{50}(KK)}$
DTG	1.0 ± 0.6	1.3
4d	0.3 ± 0.4	0.1

Table S3. The estimated binding free energies for **DTG** and **4d** bound to WT and E138K/G140A/Q148K intasomes in the stacked and non-stacked conformations.

	DTG-WT	4d-WT	DTG-KAK	4d-KAK	Notes
The probability of the complex in stacked conformer P_{com}^{S}	0.58	0.65	0.40	0.82	Occupancies from cryo-EM structures.
The probability of the complex in non-stacked conformer P_{com}^{NS}	0.42	0.35	0.60	0.18	
Binding free energy of ligand bound to IN in stacked conformer ΔG^{S}	-11.4	-11.8	-8.9	-11.0	Solved with the following two constraints
Binding free energy of ligand bound to IN in non-stacked conformer ΔG^{NS}	-11.2	-11.4	-9.2	-10.1	
overall binding free energy of ligand to IN $\Delta G_{bind} = -\frac{1}{\beta}\ln\left(e^{-\beta\Delta G^{S}} + e^{-\beta\Delta G^{NS}}\right)$	-11.7	-12.1	-9.5	-11.1	Constraint 1. (Section 1) $\Delta G_{bind} = \frac{1}{\beta}\ln(\mathrm{EC50})$
EC50(nM)	2.6	1.5	117.6	7.3	
Binding free energy difference $\Delta G^{NS} - \Delta G^{S}$	0.2	0.4	-0.2	0.9	Constraint 2. (Details in Fig 1) $\frac{1}{\beta}\ln\frac{P_{com}^{S}}{P_{com}^{NS}}$
ΔG^{S}_4d - ΔG^{S}_DTG		-0.4		**-2.1**	
ΔG^{NS}_4d - ΔG^{NS}_DTG		-0.2		**-0.9**	

REFERENCES

1. D. O. Passos, M. Li, R. Craigie, D. Lyumkis, "Retroviral integrase: Structure, mechanism, and inhibition" in (Academic Press, 2021; https://sciencedirect.com/science/article/pii/S187460472100007X), vol. 50 of *The Enzymes*, pp. 249–300.

2. G. N. Maertens, A. N. Engelman, P. Cherepanov, Structure and function of retroviral integrase. *Nat. Rev. Microbiol.* **20**, 20–34 (2022).

3. A. Engelman, K. Mizuuchi, R. Craigie, HIV-1 DNA integration: Mechanism of viral DNA cleavage and DNA strand transfer. *Cell* **67**, 1211–1221 (1991).

4. S. Hare, S. S. Gupta, E. Valkov, A. Engelman, P. Cherepanov, Retroviral intasome assembly and inhibition of DNA strand transfer. *Nature* **464**, 232–236 (2010).

5. G. N. Maertens, S. Hare, P. Cherepanov, The mechanism of retroviral integration from X-ray structures of its key intermediates. *Nature* **468**, 326–329 (2010).

6. A. Ballandras-Colas, M. Brown, N. J. Cook, T. G. Dewdney, B. Demeler, P. Cherepanov, D. Lyumkis, A. N. Engelman, Cryo-EM reveals a novel octameric integrase structure for betaretroviral intasome function. *Nature* **530**, 358–361 (2016).

7. A. Ballandras-Colas, D. P. Maskell, E. Serrao, J. Locke, P. Swuec, S. R. Jonsson, A. Kotecha, N. J. Cook, V. E. Pye, I. A. Taylor, V. Andresdottir, A. N. Engelman, A. Costa, P. Cherepanov, A supramolecular assembly mediates lentiviral DNA integration. *Science* **355**, 93–95 (2017).

8. I. K. Jóźwik, W. Li, D.-W. Zhang, D. Wong, J. Grawenhoff, A. Ballandras-Colas, S. Aiyer, P. Cherepanov, A. N. Engelman, D. Lyumkis, B-to-A transition in target DNA during retroviral integration. *Nucleic Acids Res.* **50**, 8898–8918 (2022).

9. A. Ballandras-Colas, V. Chivukula, D. T. Gruszka, Z. Shan, P. K. Singh, V. E. Pye, R. K. McLean, G. J. Bedwell, W. Li, A. Nans, N. J. Cook, H. J. Fadel, E. M. Poeschla, D. J. Griffiths, J. Vargas, I. A.

Taylor, D. Lyumkis, H. Yardimci, A. N. Engelman, P. Cherepanov, Multivalent interactions essential for lentiviral integrase function. *Nat. Commun.* **13**, 2416 (2022).

10. Z. Yin, K. Shi, S. Banerjee, K. K. Pandey, S. Bera, D. P. Grandgenett, H. Aihara, Crystal structure of the Rous sarcoma virus intasome. *Nature* **530**, 362–366 (2016).

11. V. Bhatt, K. Shi, D. J. Salamango, N. H. Moeller, K. K. Pandey, S. Bera, T. E. Bohl, F. Kurniawan, K. Orellana, W. Zhang, D. P. Grandgenett, R. S. Harris, A. C. Sundborger-Lunna, H. Aihara, Structural basis of host protein hijacking in human T-cell leukemia virus integration. *Nat. Commun.* **11**, 1–9 (2020).

12. K. K. Pandey, S. Bera, K. Shi, M. J. Rau, A. V. Oleru, J. A. J. Fitzpatrick, A. N. Engelman, H. Aihara, D. P. Grandgenett, Cryo-EM structure of the Rous sarcoma virus octameric cleaved synaptic complex intasome. *Commun. Biol.* **4**, 330 (2021).

13. M. S. Barski, J. J. Minnell, Z. Hodakova, V. E. Pye, A. Nans, P. Cherepanov, G. N. Maertens, Cryo-EM structure of the deltaretroviral intasome in complex with the PP2A regulatory subunit B56γ. *Nat. Commun.* **11**, 5043 (2020).

14. D. O. Passos, M. Li, R. Yang, S. V. Rebensburg, R. Ghirlando, Y. Jeon, N. Shkriabai, M. Kvaratskhelia, R. Craigie, D. Lyumkis, Cryo-EM structures and atomic model of the HIV-1 strand transfer complex intasome. *Science* **355**, 89–92 (2017).

15. D. O. Passos, M. Li, I. K. Jóźwik, X. Z. Zhao, D. Santos-Martins, R. Yang, S. J. Smith, Y. Jeon, S. Forli, S. H. Hughes, T. R. Burke, R. Craigie, D. Lyumkis, Structural basis for strand-transfer inhibitor binding to HIV intasomes. *Science* **367**, 810–814 (2020).

16. D. J. Hazuda, P. Felock, M. Witmer, A. Wolfe, K. Stillmock, J. A. Grobler, A. Espeseth, L. Gabryelski, W. Schleif, C. Blau, M. D. Miller, Inhibitors of strand transfer that prevent integration and inhibit HIV-1 replication in cells. *Science* **287**, 646–650 (2000).

17. A. S. Espeseth, P. Felock, A. Wolfe, M. Witmer, J. Grobler, N. Anthony, M. Egbertson, J. Y. Melamed, S. Young, T. Hamill, J. L. Cole, D. J. Hazuda, HIV-1 integrase inhibitors that compete

with the target DNA substrate define a unique strand transfer conformation for integrase. *Proc. National Acad. Sci. U.S.A.* **97**, 11244–11249 (2000).

18. N. S. Pandit, D. B. Chastain, A. M. Pallotta, M. E. Badowski, E. C. Huesgen, S. M. Michienzi, Simplifying ARV therapy in the setting of resistance. *Curr. Infect. Dis. Rep.* **21**, 38 (2019).

19. J.-L. Blanco, V. Varghese, S.-Y. Rhee, J. M. Gatell, R. W. Shafer, HIV-1 integrase inhibitor resistance and its clinical implications. *J Infect Dis* **203**, 1204–1214 (2011).

20. S. J. Smith, X. Z. Zhao, D. O. Passos, D. Lyumkis, T. R. Burke, S. H. Hughes, HIV-1 Integrase inhibitors that are active against drug-resistant integrase mutants. *Acs. Infect. Dis.* **64**, 430 (2020).

21. S. J. Smith, X. Z. Zhao, T. R. Burke Jr., S. H. Hughes, HIV-1 integrase inhibitors that are broadly effective against drug-resistant mutants. *Antimicrob. Agents Chemother.* **62**, 10.1128/aac.01035-18 (2018).

22. S. J. Smith, X. Z. Zhao, T. R. Burke, S. H. Hughes, Efficacies of Cabotegravir and Bictegravir against drug-resistant HIV-1 integrase mutants. *Retrovirology* **15**, 37 (2018).

23. S.-Y. Rhee, P. M. Grant, P. L. Tzou, G. Barrow, P. R. Harrigan, J. P. A. Ioannidis, R. W. Shafer, A systematic review of the genetic mechanisms of dolutegravir resistance. *J. Antimicrob. Chemother.* **32**, 1551 (2019).

24. J. L. Blanco, A.-G. Marcelin, C. Katlama, E. Martinez, Dolutegravir resistance mutations. *Curr. Opin. Infect. Dis.* **31**, 237–245 (2018).

25. K. Anstett, B. Brenner, T. Mesplede, M. A. Wainberg, HIV drug resistance against strand transfer integrase inhibitors. *Retrovirology* **14**, 36 (2017).

26. J. M. Coffin, HIV population dynamics in vivo: Implications for genetic variation, pathogenesis, and therapy. *Science* **267**, 483–489 (1995).

27. S. J. Smith, X. Z. Zhao, D. O. Passos, D. Lyumkis, T. R. Burke, S. H. Hughes, Integrase strand transfer inhibitors are effective anti-HIV drugs. *Viruses* **13**, 205 (2021).

28. N. J. Cook, W. Li, D. Berta, M. Badaoui, A. Ballandras-Colas, A. Nans, A. Kotecha, E. Rosta, A. N. Engelman, P. Cherepanov, Structural basis of second-generation HIV integrase inhibitor action and viral resistance. *Science* **367**, 806–810 (2020).

29. X. Z. Zhao, S. J. Smith, D. P. Maskell, M. Métifiot, V. E. Pye, K. Fesen, C. Marchand, Y. Pommier, P. Cherepanov, S. H. Hughes, T. R. Burke, HIV-1 integrase strand transfer inhibitors with reduced susceptibility to drug resistant mutant integrases. *ACS Chem. Biol.* **11**, 1074–1081 (2016).

30. X. Z. Zhao, S. J. Smith, D. P. Maskell, M. Métifiot, V. E. Pye, K. Fesen, C. Marchand, Y. Pommier, P. Cherepanov, S. H. Hughes, T. R. Burke, Structure-guided optimization of HIV integrase strand transfer inhibitors. *J. Med. Chem.* **60**, 7315–7332 (2017).

31. S. J. Smith, X. Z. Zhao, D. O. Passos, V. E. Pye, P. Cherepanov, D. Lyumkis, T. R. Burke, S. H. Hughes, HIV-1 integrase inhibitors with modifications that affect their potencies against drug resistant integrase mutants. *Acs. Infect. Dis.*. **7**, 1469–1482 (2021).

32. N. A. Margot, R. R. Ram, K. L. White, M. E. Abram, C. Callebaut, Antiviral activity of HIV-1 integrase strand-transfer inhibitors against mutants with integrase resistance-associated mutations and their frequency in treatment-naïve individuals. *J. Med. Virol.* **91**, 2188–2194 (2019).

33. K. E. Hightower, R. Wang, F. DeAnda, B. A. Johns, K. Weaver, Y. Shen, G. H. Tomberlin, H. L. Carter, T. Broderick, S. Sigethy, T. Seki, M. Kobayashi, M. R. Underwood, Dolutegravir (S/GSK1349572) exhibits significantly slower dissociation than Raltegravir and Elvitegravir from wild-type and integrase inhibitor-resistant HIV-1 integrase-DNA complexes. *Antimicrob. Agents Chemother.* **55**, 4552–4559 (2011).

34. O. Delelis, I. Malet, L. Na, L. Tchertanov, V. Calvez, A.-G. Marcelin, F. Subra, E. Deprez, J.-F. Mouscadet, The G140S mutation in HIV integrases from raltegravir-resistant patients rescues catalytic defect due to the resistance Q148H mutation. *Nucleic Acids Res.* **37**, 1193–1201 (2009).

35. M. Li, K. A. Jurado, S. Lin, A. Engelman, R. Craigie, Engineered hyperactive integrase for concerted HIV-1 DNA integration. *PLOS ONE* **9**, e105078 (2014).

36. S.-Y. Rhee, M. J. Gonzales, R. Kantor, B. J. Betts, J. Ravela, R. W. Shafer, Human immunodeficiency virus reverse transcriptase and protease sequence database. *Nucleic Acids Res.* **31**, 298–303 (2003).

37. R. W. Shafer, Rationale and uses of a public HIV drug-resistance database. *J. Infect Dis.* **194**, S51–S58 (2006).

38. A. L. Ferguson, J. K. Mann, S. Omarjee, T. Ndung'u, B. D. Walker, A. K. Chakraborty, Translating HIV sequences into quantitative fitness landscapes predicts viral vulnerabilities for rational immunogen design. *Immunity* **38**, 606–617 (2013).

39. J. P. Barton, N. Goonetilleke, T. C. Butler, B. D. Walker, A. J. McMichael, A. K. Chakraborty, Relative rate and location of intra-host HIV evolution to evade cellular immunity are predictable. *Nat. Commun.* **7**, 11660 (2016).

40. M. Figliuzzi, H. Jacquier, A. Schug, O. Tenaillon, M. Weigt, Coevolutionary landscape inference and the context-dependence of mutations in beta-lactamase TEM-1. *Mol. Biol. Evol.* **33**, 268–280 (2016).

41. T. A. Hopf, J. B. Ingraham, F. J. Poelwijk, C. P. I. Schärfe, M. Springer, C. Sander, D. S. Marks, Mutation effects predicted from sequence co-variation. *Nat. Biotechnol.* **35**, 128–135 (2017).

42. K. Shekhar, C. F. Ruberman, A. L. Ferguson, J. P. Barton, M. Kardar, A. K. Chakraborty, Spin models inferred from patient-derived viral sequence data faithfully describe HIV fitness landscapes. *Phys. Rev. E* **88**, 062705 (2013).

43. T. C. Butler, J. P. Barton, M. Kardar, A. K. Chakraborty, Identification of drug resistance mutations in HIV from constraints on natural evolution. *Phys. Rev. E* **93**, 022412 (2016).

44. A. Biswas, A. Haldane, E. Arnold, R. M. Levy, Epistasis and entrenchment of drug resistance in HIV-1 subtype B. *eLife* **8**, e50524 (2019).

45. A. Horovitz, Double-mutant cycles: A powerful tool for analyzing protein structure and function. *Fold. Des.* **1**, R121–R126 (1996).

46. T. Yoshinaga, M. Kobayashi, T. Seki, S. Miki, C. Wakasa-Morimoto, A. Suyama-Kagitani, S. Kawauchi-Miki, T. Taishi, T. Kawasuji, B. A. Johns, M. R. Underwood, E. P. Garvey, A. Sato, T. Fujiwara, Antiviral characteristics of GSK1265744, an HIV integrase inhibitor dosed orally or by long-acting injection. *Antimicrob. Agents Chemother.* **59**, 397–406 (2015).

47. J. A. Fulcher, Y. Du, T. Zhang, R. Sun, R. J. Landovitz, Emergence of integrase resistance mutations during initial therapy containing dolutegravir. *Clin. Infect. Dis.* **67**, 791–794 (2018).

48. A. M. Wensing, V. Calvez, F. Ceccherini-Silberstein, C. Charpentier, H. F. Günthard, R. Paredes, R. W. Shafer, D. D. Richman, 2022 update of the drug resistance mutations in HIV-1. *Top. Antivir. Med.* **30**, 559–574 (2022).

49. E. C. Hulme, M. A. Trevethick, Ligand binding assays at equilibrium: Validation and interpretation. *Bri. J. Pharmacol.* **161**, 1219–1237 (2010).

50. S. Hare, G. N. Maertens, P. Cherepanov, 3′-processing and strand transfer catalysed by retroviral integrasein crystallo. *EMBO J.* **31**, 3020–3028 (2012).

51. X. Z. Zhao, S. J. Smith, M. Métifiot, C. Marchand, P. L. Boyer, Y. Pommier, S. H. Hughes, T. R. Burke, 4-Amino-1-hydroxy-2-oxo-1,8-naphthyridine-containing compounds having high potency against raltegravir-resistant integrase mutants of HIV-1. *J. Med. Chem.* **57**, 5190–5202 (2014).

52. S. J. Smith, S. H. Hughes, Rapid screening of HIV reverse transcriptase and integrase inhibitors. *J. Vis. Exp.*, e51400 (2014).

53. A. Haldane, R. M. Levy, Mi3-GPU: MCMC-based inverse Ising inference on GPUs for protein covariation analysis. *Comput. Phys. Commun.* **260**, 107312 (2021).

54. R. M. Levy, A. Haldane, W. F. Flynn, Potts Hamiltonian models of protein co-variation, free energy landscapes, and evolutionary fitness. *Curr. Opin. Struc. Biol.* **43**, 55–62 (2017).

55. A. Haldane, W. F. Flynn, P. He, R. S. K. Vijayan, R. M. Levy, Structural propensities of kinase family proteins from a Potts model of residue co-variation. *Protein Sci.* **25**, 1378–1384 (2016).

56. F. Morcos, A. Pagnani, B. Lunt, A. Bertolino, D. S. Marks, C. Sander, R. Zecchina, J. N. Onuchic, T. Hwa, M. Weigt, Direct-coupling analysis of residue coevolution captures native contacts across many protein families. *Proc. National. Acad. Sci. U.S.A.* **108**, E1293–E1301 (2011).

57. D. S. Marks, T. A. Hopf, C. Sander, Protein structure prediction from sequence variation. *Nat. Biotechnol.* **30**, 1072–1080 (2012).

58. A. Biswas, A. Haldane, R. M. Levy, Limits to detecting epistasis in the fitness landscape of HIV. *PLOS ONE* **17**, e0262314 (2022).

59. T. Zhang, L. Dai, J. P. Barton, Y. Du, Y. Tan, W. Pang, A. K. Chakraborty, J. O. Lloyd-Smith, R. Sun, Predominance of positive epistasis among drug resistance-associated mutations in HIV-1 protease. *PLOS Genet.* **16**, e1009009 (2020).

60. R. H. Y. Louie, K. J. Kaczorowski, J. P. Barton, A. K. Chakraborty, M. R. McKay, Fitness landscape of the human immunodeficiency virus envelope protein that is targeted by antibodies. *Proc. Natl. Acad. Sci. U.S.A.* **115**, E564–E573 (2018).

61. W. F. Flynn, A. Haldane, B. E. Torbett, R. M. Levy, Inference of epistatic effects leading to entrenchment and drug resistance in HIV-1 protease. *Mol. Biol. Evol.* **34**, msx095 (2017).

62. C. Suloway, J. Pulokas, D. Fellmann, A. Cheng, F. Guerra, J. Quispe, S. Stagg, C. S. Potter, B. Carragher, Automated molecular microscopy: The new Leginon system. *J. Struct. Biol.* **151**, 41–60 (2005).

63. A. Cheng, C. Negro, J. F. Bruhn, W. J. Rice, S. Dallakyan, E. T. Eng, D. G. Waterman, C. S. Potter, B. Carragher, Leginon: New features and applications. *Protein Sci.* **30**, 136–150 (2020).

64. D. N. Mastronarde, Automated electron microscope tomography using robust prediction of specimen movements. *J. Struct. Biol.* **152**, 36–51 (2005).

65. D. Kimanius, L. Dong, G. Sharov, T. Nakane, S. H. W. Scheres, New tools for automated cryo-EM single-particle analysis in RELION-4.0. *Biochem. J.* **478**, 4169–4185 (2021).

66. T. Grant, N. Grigorieff, Measuring the optimal exposure for single particle cryo-EM using a 2.6 Å reconstruction of rotavirus VP6. *eLife* **4**, e06980 (2015).

67. T. Grant, A. Rohou, N. Grigorieff, cisTEM, User-friendly software for single-particle image processing. *eLife* **7**, e14874 (2018).

68. D. Tegunov, P. Cramer, Real-time cryo-electron microscopy data preprocessing with Warp. *Nat. Methods* **71**, 1–7 (2019).

69. A. Punjani, J. L. Rubinstein, D. J. Fleet, M. A. Brubaker, CryoSPARC: Algorithms for rapid unsupervised cryo-EM structure determination. *Nat. Methods* **14**, 290–296 (2017).

70. G. Harauz, M. van Heel, Exact filters for general geometry 3-dimensional reconstruction. *Optik* **73**, 146–156 (1986).

71. A. Punjani, H. Zhang, D. J. Fleet, Non-uniform refinement: Adaptive regularization improves single-particle cryo-EM reconstruction. *Nat. Methods* **17**, 1214–1221 (2020).

72. Y. Z. Tan, P. R. Baldwin, J. H. Davis, J. R. Williamson, C. S. Potter, B. Carragher, D. Lyumkis, Addressing preferred specimen orientation in single-particle cryo-EM through tilting. *Nat. Methods* **14**, 793–796 (2017).

73. P. R. Baldwin, D. Lyumkis, Non-uniformity of projection distributions attenuates resolution in Cryo-EM. *Prog. Biophys. Mol. Biol.* **150**, 160–183 (2020).

74. P. R. Baldwin, D. Lyumkis, Tools for visualizing and analyzing Fourier space sampling in Cryo-EM. *Prog. Biophys. Mol. Biol.* **160**, 53–65 (2021).

75. E. F. Pettersen, T. D. Goddard, C. C. Huang, G. S. Couch, D. M. Greenblatt, E. C. Meng, T. E. Ferrin, UCSF Chimera–A visualization system for exploratory research and analysis. *J. Comput. Chem.* **25**, 1605–1612 (2004).

76. N. W. Moriarty, R. W. Grosse-Kunstleve, P. D. Adams, Electronic Ligand Builder and Optimization Workbench (eLBOW): A tool for ligand coordinate and restraint generation. *Acta Crystallogr. Sect. D* **65**, 1074–1080 (2009).

77. P. Emsley, B. Lohkamp, W. G. Scott, K. Cowtan, Features and development of Coot. *Acta Crystallogr. D Biol. Crystallogr.* **66**, 486–501 (2010).

78. A. Brown, F. Long, R. A. Nicholls, J. Toots, P. Emsley, G. Murshudov, Tools for macromolecular model building and refinement into electron cryo-microscopy reconstructions. *Acta Crystallogr. D Biol. Crystallogr.* **71**, 136–153 (2015).

79. P. V. Afonine, B. K. Poon, R. J. Read, O. V. Sobolev, T. C. Terwilliger, A. Urzhumtsev, P. D. Adams, Real-space refinement in PHENIX for cryo-EM and crystallography. *Acta Crystallogr. Sect. D* **74**, 531–544 (2018).

80. O. Carugo, D. Bordo, How many water molecules can be detected by protein crystallography? *Acta Crystallogr. Sect D Biol. Crystallogr.* **55**, 479–483 (1999).

81. D. Liebschner, P. V. Afonine, M. L. Baker, G. Bunkóczi, V. B. Chen, T. I. Croll, B. Hintze, L.-W. Hung, S. Jain, A. J. McCoy, N. W. Moriarty, R. D. Oeffner, B. K. Poon, M. G. Prisant, R. J. Read, J. S. Richardson, D. C. Richardson, M. D. Sammito, O. V. Sobolev, D. H. Stockwell, T. C. Terwilliger, A. G. Urzhumtsev, L. L. Videau, C. J. Williams, P. D. Adams, Macromolecular structure determination using X-rays, neutrons and electrons: Recent developments in Phenix. *Acta Crystallogr. Sect. D* **75**, 861–877 (2019).

82. P. V. Afonine, B. P. Klaholz, N. W. Moriarty, B. K. Poon, O. V. Sobolev, T. C. Terwilliger, P. D. Adams, A. Urzhumtsev, New tools for the analysis and validation of cryo-EM maps and atomic models. *Acta Crystallogr. Sect. D Struct. Biol.* **74**, 814–840 (2018).

83. C. J. Williams, J. J. Headd, N. W. Moriarty, M. G. Prisant, L. L. Videau, L. N. Deis, V. Verma, D. A. Keedy, B. J. Hintze, V. B. Chen, S. Jain, S. M. Lewis, W. B. Arendall, J. Snoeyink, P. D. Adams, S. C. Lovell, J. S. Richardson, D. C. Richardson, MolProbity: More and better reference data for improved all-atom structure validation. *Protein Sci.* **27**, 293–315 (2018).

84. L. Wang, Y. Wu, Y. Deng, B. Kim, L. Pierce, G. Krilov, D. Lupyan, S. Robinson, M. K. Dahlgren, J. Greenwood, D. L. Romero, C. Masse, J. L. Knight, T. Steinbrecher, T. Beuming, W. Damm, E. Harder, W. Sherman, M. Brewer, R. Wester, M. Murcko, L. Frye, R. Farid, T. Lin, D. L. Mobley, W. L. Jorgensen, B. J. Berne, R. A. Friesner, R. Abel, Accurate and reliable prediction of relative ligand binding potency in prospective drug discovery by way of a modern free-energy calculation protocol and force field. *J. Am. Chem. Soc.* **137**, 2695–2703 (2015).

85. K. Roos, C. Wu, W. Damm, M. Reboul, J. M. Stevenson, C. Lu, M. K. Dahlgren, S. Mondal, W. Chen, L. Wang, R. Abel, R. A. Friesner, E. D. Harder, OPLS3e: Extending force field coverage for drug-like small molecules. *J. Chem. Theory Comput.* **15**, 1863–1874 (2019).

86. H. J. C. Berendsen, J. P. M. Postma, W. F. van Gunsteren, J. Hermans, Interaction models for water in relation to protein hydration intermolecular force. *Jerus Symposia Quantum Chem. Biochem.* (1981), vol. 14.

87. C. H. Bennett, Efficient estimation of free energy differences from Monte Carlo data. *J. Comput. Phys.* **22**, 245–268 (1976).

88. S. Aiyer, C. Zhang, P. R. Baldwin, D. Lyumkis, Evaluating local and directional resolution of cryo-EM density maps. *Methods Mol. Biol.* **2215**, 161–187 (2021).
